# Welcome editorial by the new *CKJ* Editor-in-Chief: Facing the future of *CKJ* with enthusiasm!

**DOI:** 10.1093/ckj/sfac121

**Published:** 2022-05-30

**Authors:** María José Soler

**Affiliations:** Hospital Universitari Vall d'Hebron, Autonomous University of Barcelona, Barcelona, Spain

## BACKGROUND

In early 2008, the ERA-EDTA launched *NDT Plus* under the Editorship of Norbert Lameire with the aim of providing an educational and training resource focusing on postgraduate clinical education and topics of interest to the clinical nephrologist [1]. Four years later, in February 2012, *NDT Plus* evolved into the *Clinical Kidney Journal* (*CKJ*) under the leadership of Alain Meyrier [2]. The new *CKJ* flourished and accomplished its mission: the quality of the journal clearly improved, resulting in a rejection rate of 60% [3]. In 2015, a new chapter of the journal started when Alberto Ortiz was elected as *CKJ* Editor-in-Chief. A subheading was added to *CKJ* emphasizing its new focus: *Clinical and Translational Nephrology* [3]. The vision was further expanded in a series of high-quality articles on translational nephrology [4]. The journal became open access (ERA members get a 33% discount on open access fees), monthly and online-only, and fully searchable and accessible through PubMed. The types of manuscripts were streamlined to Editorial Comments, *CKJ* Reviews, Original Articles, Exceptional Cases and Letters to the Editor [4].

**FIGURE 1:**
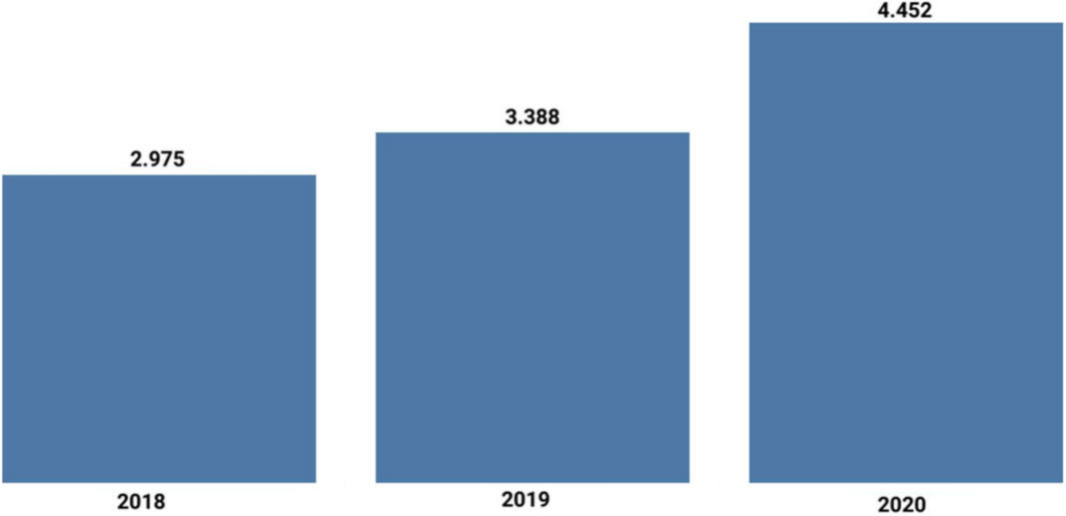
Impact factor trend.

**FIGURE 2:**
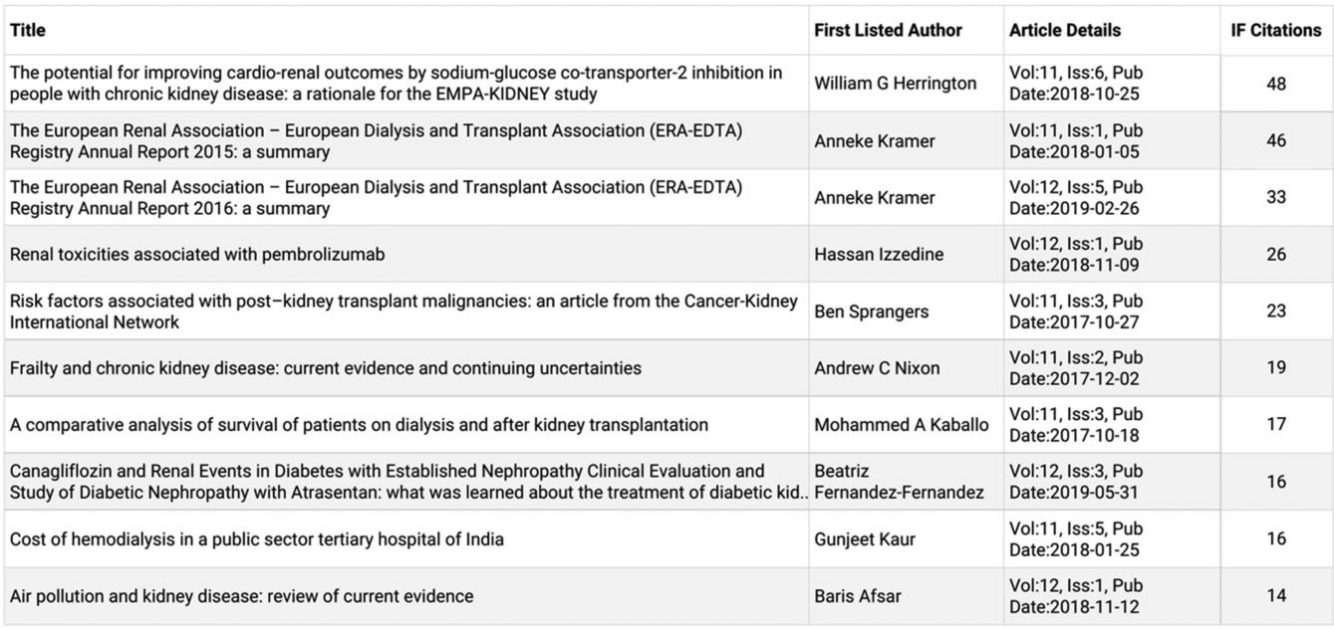
Which articles received the most citations, contributing to the 2020 impact factor?

Over the past 5 years, the acceptance rate has gradually decreased, to ∼22% in early 2022. The types of manuscripts published shifted towards an increase in high-quality *CKJ* Reviews and Original Articles. These changes were well-received by the nephrological community, and the number of citations per year exponentially increased. Under the direction of Alberto Ortiz, *CKJ* was accepted for indexing in the new Emerging Sources Citation Index (ESCI) database from Clarivate Analytics (previously Thomson Reuters) in 2016. *CKJ* received its first journal impact factor (JIF) in 2019 [5]. The Impact Factor went up to 4.452, ranking *CKJ* in the first quartile of urology and nephrology journals [5] (Figures 1 and 2). Concretely, *CKJ* is the 9th in the ranking of overall nephrology journals and the 6th ranked nephrology journal among journals that mainly publish original research [5]. Clarivate even ranked *CKJ* as the top open-access nephrology journal.

## *CKJ*—A GOOD HOME FOR YOUR PUBLICATION

**FIGURE 3:**
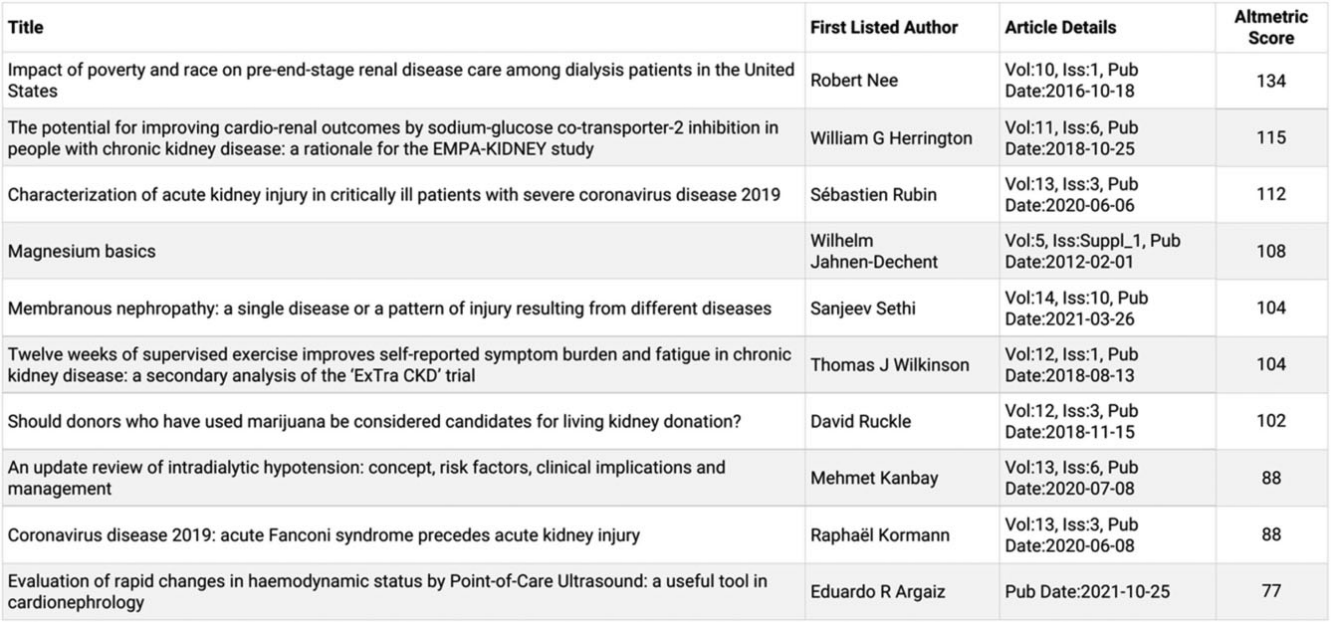
Top 10 articles by all-time Altmetrics score. Which articles have received most attention?

All figures published in *CKJ* are redesigned by a professional medical illustrator before publication. *CKJ* authors are also encouraged to submit a graphical abstract or video/audio abstract with their *CKJ* publication. Our authors can register for and submit their ORCID identities alongside their papers. By registering for an ORCID ID, authors can be sure that their work will be properly attributed and referenced, and made easily discoverable. *CKJ* wants to publish its accepted scientific work as quickly as possible and therefore publishes the accepted, unedited manuscript online within 48 h after the author license has been signed. The accepted manuscript is then replaced by the final, typeset proof within 5 to 6 weeks. *CKJ* is also very active on social media and posts articles on the *CKJ* Twitter account (@*CKJ*social) daily. I am planning to further increase *CKJ*'s visibility on social media (Facebook, LinkedIn, Twitter and Instagram) and to introduce monthly Tweetorials. In this respect, each online *CKJ* publication also shows its individual Altmetrics score, which is the amount of attention the article has received online, on social media and from news sites. A summary of the highest all-time Altmetrics-scoring papers is shown in Figure 3.

## THE *CKJ* JOURNAL CLUB

**FIGURE 4:**
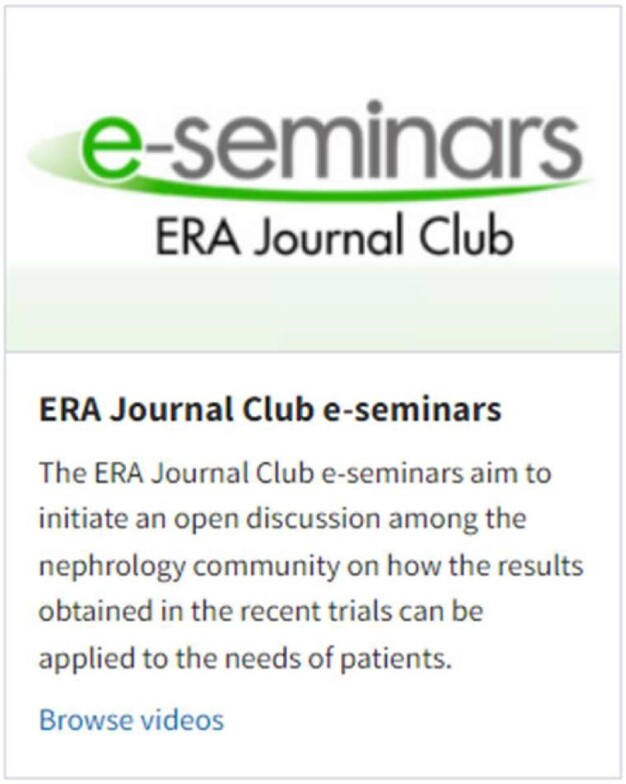
The 2022 ERA Journal Club, previously the *CKJ* Journal Club.

After the global COVID-19 breakthrough, webinars were widely implemented in different fields of medicine, mainly for educational purposes. Nephrology could not be left behind and *CKJ* therefore launched the *CKJ* Journal Club in October 2020 and has continued since then. Each month, a high-impact article is discussed in a webinar with the authors, external expert panelists and Will Herrington or Kate Stevens and Jennifer Lees as the moderators. During these webinars, the audience can ask live questions to the speakers, giving them the opportunity to directly interact with the author. Since 2022, the *CKJ* Journal Club has become the ERA Journal Club, now also discussing *NDT* publications (Figure 4).

## *CKJ* COLLABORATES WITH OTHER JOURNALS

**FIGURE 5:**
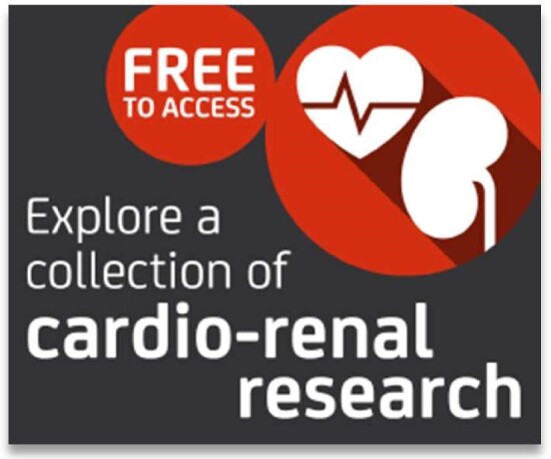
The cardio-renal collection including articles from *NDT/CKJ* and the ESC journals published by OUP.

In 2021, *CKJ* continued the cardio-renal collection, a virtual journal including cardio-renal medicine articles from *NDT, CKJ* and the European Society of Cardiology journals published by OUP (Figure 5). *CKJ* will soon also launch a new virtual collection including articles from *NDT* and *CKJ* and *Age and Ageing*, the official journal of the British Geriatrics Society (JIF of 10.668). After all, many kidney patients are also elderly.

During my term as *CKJ* Editor-in-Chief, I am also planning to work closely together with the *NDT* Editor-in-Chief and ERA Council to achieve harmony between the ‘brother’ journals *NDT* and *CKJ*.

## FUTURE PERSPECTIVES

Although Alberto Ortiz and the previous Editors-in-Chief have done a fantastic job, and I know that I have some very big shoes to fill, I continuously want to explore new ways to further improve *CKJ*. We will submit our Medline application in July 2022, and the final decision will be announced in July 2023. If *CKJ* is accepted, then all *CKJ* publications will be retrievable in PubMed immediately after their advance access publication (as is the case for *NDT*). Further items on the agenda are the introduction of podcasts, and plain-English summaries in our publications, which will also be very useful for social media purposes.

**FIGURE 6:**
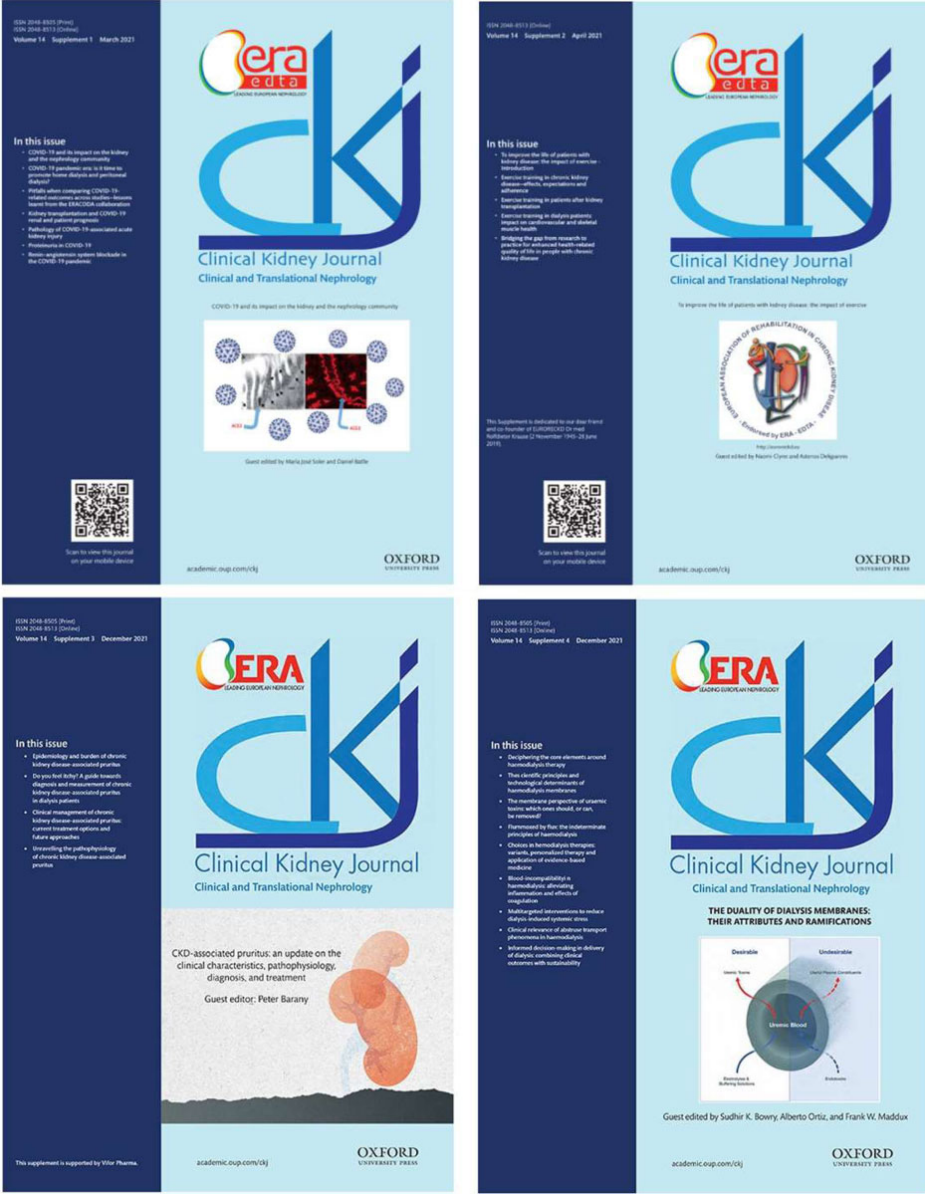
*CKJ* Supplements published in 2021.

*CKJ* published four Supplements in 2021: ‘COVID-19 and its impact on the kidney and the nephrology community’, ‘To improve the life of patients with kidney disease: the impact of exercise’, ‘CKD-associated pruritus: an update on the clinical characteristics, pathophysiology, diagnosis and treatment’ and ‘The duality of dialysis membranes: their attributes and ramifications’ (see Figure 6). Since several Supplement articles are very well cited, I will continue to publish four to five high-quality Supplements in 2022.

## REVIEWERS AND EDITORS—THE BACKBONE OF OUR JOURNAL

**FIGURE 7:**
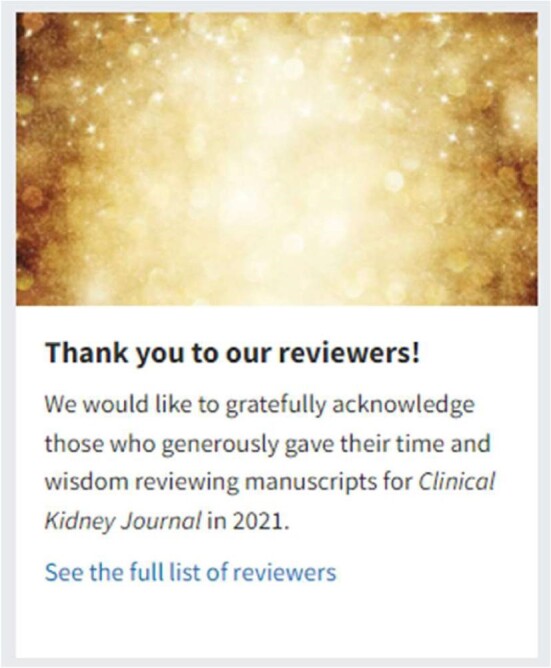
Thank you to our reviewers’ message on the *CKJ* homepage.

I am well aware that the journal's success largely depends on the invaluable support of our reviewers and editors. Currently, we offer our reviewers the possibility to get recognition for their reviews via Publons. A special ‘thank you’ message for the *CKJ* reviewers has also been posted on the *CKJ* homepage (Figure 7), and I will further investigate how to appropriately reward our reviewers in 2022. Following the new scope of the journal, I am planning to evolve the journal by also modifying the editorial board. There will be five new associate editors in addition to the other theme editors. Together with these five associate editors (Mario Cozzolino, Francesca Mallamaci, Kate Stevens, Roser Torra and Christoph Wanner) there will be (bi)-weekly editorial meetings to decide on the outcome of certain *CKJ* submissions and to decide on the journal's strategy and further developments. I am further planning to welcome additional gender-balanced editorial board members and statistical experts to contribute to the scientific level of the journal.

## EDITORIAL ASSISTANCE

I am most grateful to the past Editors-in-Chief, dedicated editorial board team and reviewers that have made *CKJ* the high-quality first quartile journal it has become. But some aspects of *CKJ* will not change… In that sense, the excellent editorial assistance will continue to be provided by Caroline Vinck.

I expect that the new format of having weekly editorial meetings, in combination with the excellent and hard work of our authors, reviewers, editorial board members and editorial office, will result in an enjoyable, thought-provoking journal focused on educational nephrology, all aspects of clinical diseases, nephropathology, interventional nephrology, clinical trials and research in nephrology, with the main goals to promote ‘nephrology’ as a specialty and to improve the care for our patients.

Over the next 3 years, I will improve or maintain *CKJ*'s impact factor and try to deliver to our readers one of the best clinical nephrology journals.

Last but not least, I would like to thank the ERA Council for giving me the thrust and support to take on this important task. I am very enthusiastic and excited to start this new phase in my life!
